# „Auf einmal merke ich, dass ich wach werde …“ – Awareness aus Sicht einer Betroffenen

**DOI:** 10.1007/s00101-025-01612-8

**Published:** 2025-11-18

**Authors:** Rainer Thomas, Kerstin Heinken

**Affiliations:** Schiffer Campus, Klinik für Anästhesiologie, Universitätsmedizin Frankfurt, Schulstraße 31, 60594 Frankfurt, Deutschland

## Anamnese, Diagnose, Verlauf

Eine 36-jährige Patientin stellt sich zu einem mittelgroßen chirurgischen Eingriff vor. Es bestehen keine Vorerkrankungen, ebenso wenig Allergien und es wird keine Dauermedikation eingenommen. Bisherige Anästhesien waren stets ohne Auffälligkeiten verlaufen. Die Patientin selbst ist Weiterbildungsassistentin in der Anästhesie in einem anderen Klinikum. Im Folgenden erleben wir den Ablauf der Anästhesie aus Sicht der Patientin und der betreuenden Anästhesistin.

**Patientin:** *„Vermutlich unterzieht sich niemand gerne einem operativen Eingriff. Deshalb freue ich mich, dass ich die bei mir geplante Operation bei meinem ehemaligen Arbeitgeber durchführen lassen kann. So kann ich mir auch meine Anästhesistin selbst aussuchen und begebe mich vertrauensvoll in die Hände meiner Freundin und Kollegin, einer erfahrenen Fachärztin für Anästhesie.“*

**Ärztin:** *„Meine erste Patientin am heutigen Tag ist eine besondere, nämlich eine Freundin und ehemalige Kollegin. Deshalb möchten wir es bei dieser für drei Stunden angesetzten Operation in Bauchlage besonders gut, es ihr besonders angenehm machen.“*

**Patientin:** *„Nach Ankunft in der Einleitung und einem kurzen Pläuschchen sprechen wir noch darüber, dass ich nach der Einleitung einen zweiten Zugang bekomme. Mir ist das wichtig, da ich weiß, dass ich eine totale intravenöse Anästhesie (TIVA) bekommen werde. Mit einem guten Gefühl und leichtem Brennen in der Vene vom Propofol schlafe ich ein.“*

**Ärztin:** *„Leider ist unser einziges EEG-Gerät heute in einem anderen OP im Einsatz, daher müssen wir trotz Durchführung einer TIVA ohne dieses Hilfsmittel auskommen. Sorge habe ich deshalb jedoch keine, da die vorangegangene Narkose einige Monate zuvor ohne jegliche Auffälligkeiten verlaufen ist. Nach problemloser Narkoseeinleitung mit 0,35 µg/kgKG und min Remifentanil, 250 mg Propofol und 30 mg Atracurium und anschließender Lagerung erhalte ich die Anästhesie durch kontinuierliche Gabe von 7 mg/kgKG und h Propofol und 0,3 µg/kgKG und min Remifentanil aufrecht.“*

**Patientin:** *„Auf einmal merke ich, dass ich wach werde. Ich spüre, dass ich noch im OP bin, kann aber nicht einschätzen, wie weit fortgeschritten der Eingriff ist oder ob er bereits beendet wurde. Ich denke bewusst darüber nach, was ich tun kann, um auf mich aufmerksam zu machen. Da ich nicht weiß, ob ich noch relaxiert bin, versuche ich mich zu bewegen. Ich bin nicht in der Lage, ein Körperteil bewusst anzusteuern, offensichtlich sind meine Bemühungen jedoch erfolgreich, denn sofort höre ich die Chirurgin: „Sie ist wach!“. Kurz danach schlafe ich auch schon wieder ein.“*

**Abb. 1:**
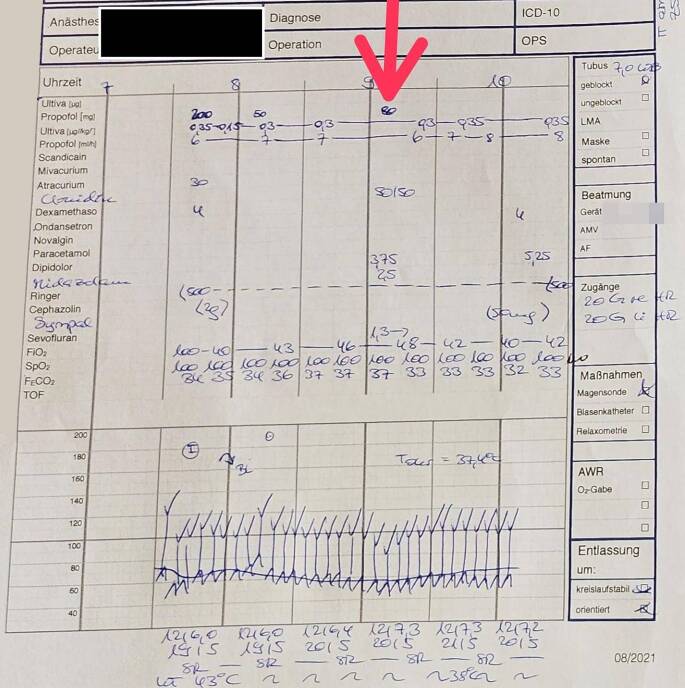
Anästhesieprotokoll, *Pfeil* markiert den Zeitpunkt der Awareness

**Ärztin:** *„Etwa eine Stunde nach Operationsbeginn hebe ich für eine Lagerungskontrolle den Kopf leicht aus dem Schaumkissen, um Augen und Nase über den Spiegel besser einsehen zu können. Nur Sekunden später bekomme ich von der Operateurin die Rückmeldung, dass die Patientin das Bein anzieht, und ich selbst bemerke ein aktives Anheben des Kopfes. Ich reagiere sofort und verabreiche 80 mg Propofol, 3,75 mg Piritramid, 2,5 mg Midazolam und 150 µg Clonidin. Zudem öffne ich den Sevofluran-Vapor, bis eine endtidale Konzentration von 1,3 Vol.-% erreicht ist. Auf dem Monitor gibt es keinerlei Hinweise auf eine stattgehabte Awareness, auch nicht bei Durchsicht der Werte im Trend. Weder ansteigende Blutdruckwerte noch eine Veränderung der Herzfrequenz sind zu sehen. Entsprechend ist es auch auf dem Anästhesieprotokoll dokumentiert (*Abb. 1*). Auch finden sich am venösen Zugang, über welchen das Propofol verabreicht wird, keine Anzeichen für ein Paravasat. Ebenso wenig gibt es eine hämodynamische Reaktion auf unsere Interventionen, und wir hoffen, dass es nicht zu einer erinnerlichen Awareness gekommen ist.“*

**Patientin:** „Als ich wieder wach werde, liege ich bereits im Aufwachraum. Meine ehemaligen Chefs stehen an meinem Bett, ebenso die Kollegen aus der Pflege. Im Halbschlaf teile ich noch halb lallend mit, dass ich während der Operation wach war. Die Frage, ob ich währenddessen Schmerzen hatte, kann ich glücklicherweise verneinen. Und so ruhe ich mich noch ein wenig aus und werde dann entlassen. Rückblickend war es für mich ein merkwürdiges Gefühl, bei Bewusstsein zu sein, jedoch nicht zu wissen, ob die Operation bereits vorbei oder noch im Gange ist. Da ich keine Schmerzen hatte, und meine Awareness schnell erkannt wurde, habe ich das Ganze nicht als traumatisierend empfunden. Alles im Leben ist für etwas gut, und so habe ich aus meinem eigenen Erlebnis gelernt, wie wichtig es ist, auch mit schlafenden Patient*innen zu sprechen und beruhigend für sie da zu sein. Denn ich kann nie ganz sicher sein, was meine Patient*innen während einer Anästhesie alles mitbekommen.“

**Ärztin:** *„Was alles passieren kann, wenn man eigentlich alles besonders korrekt machen will. Besonders eindrücklich war für mich, dass eine Awareness aufgetreten ist, obgleich sich diese mit keinerlei vegetativen Veränderungen angedeutet hat. Zukünftig werde ich vermutlich noch mehr an diese seltene Komplikation denken. Immerhin: Die Leitung unserer Praxis hat bereits reagiert und weitere EEG-Geräte angeschafft.“*

## Diskussion

Die akzidentelle intraoperative Wachheit oder Awareness ist eine seltene Komplikation. Die Inzidenz wird mit etwa 0,1 %–0,2 % pro Allgemeinanästhesie in unselektierten Patientenkollektiven angegeben [1]. Bestimmte Eingriffe sind mit einem erhöhten Risiko für eine intraoperative Wachheit vergesellschaftet. Hierzu zählen v. a. geburtshilfliche, kardiochirurgische und Notfalleingriffe [2]. Relevante perioperative Risikofaktoren sind eine prolongierte Atemwegssicherung, die Verwendung von Muskelrelaxanzien, flache Anästhesieführung oder Unterbrechung der Zufuhr von Hypnotika beim Transfer von Patient*innen sowie womöglich auch die Durchführung einer Narkose als TIVA [3, 4]. Patientenseitige Risikofaktoren stellen vor allen Dingen vorangegangene Ereignisse intraoperativer Wachheit, junges Alter und weibliches Geschlecht dar [4–6].

Patient*innen, die eine unbeabsichtigte intraoperative Wachheit erlebt haben, schildern diese oftmals als sehr unangenehm bis hin zur Agonie [7]. Entsprechend dramatisch können die Folgen sein, das Auftreten einer posttraumatischen Belastungsstörung nach einem solchen Ereignis wurde wiederholt beschrieben [8].

Zur Vermeidung von intraoperativen Wachheitserlebnissen wird empfohlen, im Rahmen einer balancierten Anästhesie eine minimalalveoläre Konzentration (MAC) von 0,7 nicht zu unterschreiten [9]. Zudem sollte stets ein neuromuskuläres Monitoring eingesetzt werden, wenn Muskelrelaxanzien zum Einsatz kommen, um Restblockaden zuverlässig zu detektieren. Zudem sollten Muskelrelaxanzien nur eingesetzt werden, wenn tatsächlich notwendig, da ihr Einsatz die Inzidenz einer Awareness erhöhen kann [3]. Ob die perioperative Gabe von Benzodiazepinen eine protektive Wirkung hinsichtlich einer unbeabsichtigten Wachheit hat, wird kontrovers diskutiert [10, 11].

Bezüglich des Einsatzes eines prozessierten Elektroenzephalogramms (pEEG) liegen heterogene Daten vor. Insgesamt kann davon ausgegangen werden, dass zumindest im Rahmen einer TIVA und bei hohem perioperativen Risiko der Einsatz von pEEG-Monitoren sinnvoll und zu fordern ist [2, 12]. Dies gilt insbesondere dann, wenn während eines Eingriffs der venöse Zugang lagerungsbedingt nicht regelhaft im Blick gehalten und somit die sichere Infusion von Hypnotika in das Gefäßsystem nicht überwacht werden kann. Generell gilt, dass sowohl beim Einsatz eines pEEG-Monitors als auch bei der Überwachung der endtidalen Konzentration von volatilen Anästhetika im Rahmen einer balancierten Anästhesie sinnvoll gewählte Alarmgrenzen eingestellt werden sollten. Hiermit kann ein akzidentelles Nichtwahrnehmen einer möglichen Awareness durch die betreuenden Anästhesist*innen vermieden werden.

Kommt es während einer Anästhesie zu einer vermuteten unbeabsichtigten Wachheit, so sollte umgehend die Narkose vertieft und mit beruhigender Ansprache auf die Patient*in eingewirkt werden. Die Gabe von Benzodiazepinen induziert lediglich eine ante-, jedoch keine retrograde Amnesie, kann somit das Erlebte nicht unvergessen machen und sollte keinesfalls das Beruhigen der Patient*in verzögern [11]. Zudem müssen nach einem derartigen Vorkommnis wiederholt standardisiert postoperative Interviews durchgeführt werden, um eine etwaige Awareness als solche zu identifizieren und betroffene Patient*innen adäquat zu betreuen [13]. Stets sollte in einem solchen Fall auch psychologische Hilfe angeboten werden.

Für den geschilderten Fall kann festgehalten werden, dass der Zwischenfall für die Patientin durch mehrere glückliche Umstände glimpflich verlaufen ist. Zunächst ist die Betroffene eine sehr resiliente anästhesiologische Kollegin, die keinerlei Schmerzen während der Wachheitsphase hatte. Zudem gelang es ihr, sich bemerkbar zu machen, was bei tieferer Muskelrelaxierung vermutlich nicht möglich gewesen wäre. Diskutabel ist der sicherlich wohlmeinende, jedoch etwas polypragmatische Einsatz von fünf verschiedenen Substanzen zum Zeitpunkt der Awareness. Sinnvoller wäre neben eine Vertiefung der Anästhesie mittels Propofol und einer adäquaten Analgesie eine beruhigende und erklärende Ansprache an die Patientin gewesen. Ob der Einsatz eines pEEG die Wachheit in diesem Fall hätte verhindern können, bleibt Gegenstand von Spekulationen, sicherlich wäre jedoch in der vorliegenden Konstellation (TIVA, weibliches Geschlecht und junges Patientenalter) eine EEG-Überwachung der Anästhesie ratsam gewesen. Eine exakte Ursache für die Wachheit ließ sich letztlich nicht feststellen. Es fanden sich keinerlei Anzeichen für ein Paravasat oder eine durch Stauung verursachte mangelnde Infusion des Propofols. Da eine einprozentige Lösung Verwendung fand, auf die der Perfusor, inklusive Patientengewicht, korrekt programmiert war, und im betreffenden Operationsbereich ansonsten nur eine zweiprozentige Dosierung zur Verfügung steht, scheidet auch die akzidentelle Gabe einer zu niedrigen Dosis aus.

## Fazit für die Praxis

- Der geschilderte Fall zeigt, dass eine unbeabsichtigte intraoperative Wachheit jederzeit im anästhesiologischen Alltag auftreten und mitunter auch nur schwerlich antizipiert werden kann.
- Gerade für spezielle Risikokonstellation ist deshalb die Verwendung eines pEEG dringend anzuraten.
- Generell sollten Anästhesist*innen ein Bewusstsein für die mögliche Gefahr einer unbeabsichtigten Wachheit besitzen und im klinischen Alltag diesbezüglich stets alert sein.
